# Acetylation- and ubiquitination-regulated SFMBT2 acts as a tumor suppressor in clear cell renal cell carcinoma

**DOI:** 10.1186/s13062-024-00480-3

**Published:** 2024-05-11

**Authors:** Qingpeng Xie, Bin Hu, Haosong Li

**Affiliations:** 1 Department of Urology, Liaoning Cancer Hospital and Institute, Cancer Hospital of China Medical University, No. 44 Xiaoheyan Road, Dadong District, Shenyang, 110042 Liaoning China; 2 Department of Pediatrics, Central Hospital Affiliated to Shenyang Medical College, Shenyang, Liaoning China

**Keywords:** Clear cell renal cell carcinoma, Scm-like with four MBT domains protein 2, Growth, Metastasis, Ubiquitination, Acetylation

## Abstract

**Background:**

Clear cell renal cell carcinoma (RCC) is the most common kidney tumor. The analysis from medical database showed that Scm-like with four MBT domains protein 2 (SFMBT2) was decreased in advanced clear cell RCC cases, and its downregulation was associated with the poor prognosis. This study aims to investigate the role of SFMBT2 in clear cell RCC.

**Methods:**

The expression of SFMBT2 in clear cell RCC specimens were determined by immunohistochemistry staining and western blot. The overexpression and knockdown of SFMBT2 was realized by infection of lentivirus loaded with SFMBT2 coding sequence or silencing fragment in 786-O and 769-P cells, and its effects on proliferation and metastasis were assessed by MTT, colony formation, flow cytometry, wound healing, transwell assay, xenograft and metastasis experiments in nude mice. The interaction of SFMBT2 with histone deacetylase 3 (HDAC3) and seven in absentia homolog 1 (SIAH1) was confirmed by co-immunoprecipitation.

**Results:**

In our study, SFMBT2 exhibited lower expression in clear cell RCC specimens with advanced stages than those with early stages. Overexpression of SFMBT2 inhibited the growth and metastasis of clear cell RCC cells, 786-O and 769-P, in vitro and in vivo, and its silencing displayed opposites effects. HDAC3 led to deacetylation of SFMBT2, and the HDAC3 inhibitor-induced acetylation prevented SFMBT2 from SIAH1-mediated ubiquitination modification and proteasome degradation. K687 in SFMBT2 protein molecule may be the key site for acetylation and ubiquitination.

**Conclusions:**

SFMBT2 exerted an anti-tumor role in clear cell RCC cells, and HDAC3-mediated deacetylation promoted SIAH1-controlled ubiquitination of SFMBT2. SFMBT2 may be considered as a novel clinical diagnostic marker and/or therapeutic target of clear cell RCC, and crosstalk between its post-translational modifications may provide novel insights for agent development.

**Supplementary Information:**

The online version contains supplementary material available at 10.1186/s13062-024-00480-3.

## Introduction

Renal cell carcinoma (RCC) is the seventh common tumor in the world, with 350,000 new cases and 140,000 deaths per year [1, 2]. RCC originates from the renal parenchymal urinary tubular epithelial system, and the detailed pathogenesis has not been completely elucidated. Several risk factors have been identified, such as hypertension, smoking and obesity [3–5]. RCC contains some heterogeneous cancer subtypes with various genetic and molecular characteristics, and histological alterations. The most common types are clear-cell, papillary and chromophobe RCC, and account for 85–90% of all renal malignancies [6]. Clear cell RCC is the most common subtype, accounting for 70–80%, and it is named because that the abundant lipid in the cytoplasm is dissolved during histological preparation methods and leaves a clear cytoplasm [7]. Due to latent early symptoms, the prognosis of patients with advanced RCC is poor. Clear cell RCC has a worse survival than papillary RCC and chromophobe RCC [8]. It will be beneficial for RCC patients to developing novel molecular markers and targets.

Scm-like with four MBT domains protein 2 (SFMBT2) is a member of polycomb group proteins (PcGs) family, which act as chromatin regulators to be involved in diverse biological processes such as gene transcription, DNA repair, cell differentiation, embryonic development and tumor initiation and progression [9]. SFMBT2 was demonstrated to suppress metastasis of prostate cancer cells, and SFMBT2-silenced tumor cells enhanced M2 polarization of macrophages in microenvironment [10, 11]. The roles of SFMBT2 in RCC have not been elucidated. The medical database UALCAN (https://ualcan.path.uab.edu/) showed that the SFMBT2 expression was higher in clear cell RCC specimens than normal kidney tissues, but it was lower in advanced specimens. Moreover, the analysis from another medical database Kaplan–Meier Plotter (http://kmplot.com/analysis/index.php?p=background) revealed that the clear cell RCC patients with downregulated SFMBT2 expression have poorer survival. These evidences suggested that SFMBT2 may be involved in occurrence or/and development of clear cell RCC.

SFMBT2 is reported to repress the transcription of homeobox B13, matrix metalloproteinase 2 (MMP2) and MMP9 [10, 12]. However, regulation of SFMBT2 has rarely been reported. A recent paper reported that SFMBT2 bound to histone deacetylase 3 (HDAC3) [10], which mediated the deacetylation of lysine residue in histone or non-histone. Acetylation is a common post-translational modification, and it is catalyzed by lysine acetyltransferases (KATs), and removed by lysine deacetylases (KDACs) [13]. Histone acetylation generally promotes transcription and nucleosome remodeling [14, 15], and functions of non-histone acetylation is still under investigation. Possible functions include altering the protein’s conformation, stability, hydrophobicity or localization, or blocking its capacity to accept other post-translational modifications [16, 17]. For instance, HDAC3 accelerated ubiquitination and degradation of WISP2 by reducing its acetylation level [18]. It needs to illustrate if HDAC3 affects SFMBT2.

Ubiquitination is another important post-translational modification of proteins, and the dysregulation of ubiquitination-mediated protein degradation contributes to multiple human diseases, including tumorigenesis [19, 20]. Ubiquitin is activated by ubiquitin-activating enzyme (E1), and transferred by ubiquitin-conjugating enzyme (E2) to a specific substrate along with E3 ligase, which is responsible for the substrate specificity [19]. The data from bioinformatics websites BioGRID (https://thebiogrid.org/) and HitPredict (http://www.hitpredict.org/) exhibited that SFMBT2 potentially bind to seven in absentia homolog 1 (SIAH1), an E3 ubiquitin ligase. SIAH1 was demonstrated to mediate the ubiquitination and degradation of β-catenin, ZEB1, Axin and Akt, which played key roles in tumor progression [19]. It is unknown weather SIAH1 catalyze the ubiquitination of SFMBT2.

In this study, the interaction among SFMBT2, SIAH1 and HDAC3 was explored, and the function of SFMBT2 in clear cell RCC cells was verified.

## Material and methods

### Clinical specimens

Sixty-nine cases of clear cell RCC specimens were collected from January 2022 to June 2023 in Cancer Hospital of China Medical University. The clear cell RCC was diagnosed by pathological staining. The collection and detection of specimens were performed according to Declaration of Helsinki, and the informed consent has been obtained from every patient. The experimental procedure was approved by Ethics Committee of in Cancer Hospital of China Medical University (approval number: 20230450).

### Construction of lentivirus and plasmid vectors

In order to change the expression of SFMBT2, its encoding sequence was inserted into lentivirus vector pLVX-IRES-puro, and the short hairpin RNA (shRNA) targeting SFMBT2 was inserted into lentivirus vector pLVX-shRNA1. The lentivirus vectors were packed using tool cells.

To investigate the function details of SFMBT2, its wild type (WT) encoding sequence and acetylation/ubiquitination-deficient mutant sequences (K504A, K505A, K687A) were inserted into pcDNA3.1 vector, respectively.

The encoding sequences of HDAC3 and SIAH1 were cloned into pcDNA3.1 vector, respectively, to overexpress these two genes.

The shRNA sequences were shown below:

- shSFMBT2-1: 5′-CCGGGTTGGTGTCAAGAGAATAAATTCAAGAGATTTATTCTCTTGACACCAACCTTTTTT-3′
- shSFMBT2-2: 5′-CCGGGATAGTTAGTGTGATTGAAATTCAAGAGATTTCAATCACACTAACTATCCTTTTTT-3′
- shNC: 5′-CCGTTCTCCGAACGTGTCACGTTTCAAGAGAACGTGACACGTTCGGAGAATTTTTT-3′

### Cell culture and treatment

Human renal cell carcinoma cell lines, 786-O and 769-P, were purchased from iCell (Shanghai, China), and culture with RPMI-1640 (Solarbio, Beijing, China) supplemented with 10% fetal bovine serum (FBS) (TIANHANG, Huzhou, Zhejiang, China) in a humid incubator with 37 °C and 5% CO_2_.

The cells were incubated with lentivirus for 48 h, and treated with 1.5 μg/ml puromycin (Solarbio, Beijing, China). When the blank cells all dead, the survived cells with lentivirus infection were continuously cultured as the SFMBT2-stably-overexpressed or silenced cells.

The transfection was performed using plasmid and Lipofectamine 3000 reagent (Invitrogen Corporation, Carlsbad, CA, USA) in serum-free medium.

To abolish the function of HDAC3, a specific inhibitor RGFP966 (10 μM) (Macklin, Shanghai, China) was used to treat cells for 48 h.

To block the protein synthesis, an inhibitor cycloheximide (CHX) (10 μg/ml) (MedChemExpres, Monmouth Junction, NJ, USA) was applied for 0 h, 1 h, 2 h or 4 h.

A proteasome inhibitor MG132 (10 μM) (Macklin, Shanghai, China) was administrated for 6 h to intercept the protein degradation.

### Western blot

The protein was extracted with lysis buffer (Beyotime, Shanghai, China) supplemented with 1 mM PMSF. After concentration determination, the protein underwent SDS-PAGE, and the gel concentration was set according to the protein molecule size. After electrophoresis, the protein in gel was transferred onto PVDF membrane (Abcam, Cambridge, MA, USA), which was blocked for 60 min to block the non-specific antigens, and incubated with following primary antibodies at 4 °C overnight: rabbit anti-SFMBT2 (1:1000; cat no. 25256-1-AP, Proteintech, Wuhan, Hubei, China), rabbit anti-HDAC3 (1:5000; cat no. ab32369, Abcam, Cambridge, MA, USA), mouse anti-SIAH1 (1:300; cat no. sc-81785, Santa Cruz, CA, USA), mouse anti-β-actin (1:1000; cat no. sc-47778, Santa Cruz, CA, USA). The PVDF membrane was rinsed with TBST, and incubated with goat anti-rabbit or anti-mouse IgG labeled with horse radish peroxidase (HRP) (1:5000; Beyotime, Shanghai, China) at 37 °C for 45 min. Finally, the membrane interacted with ECL reagent, and the signal exposure was performed in the dark. β-actin served as the internal control.

### Co-immunoprecipitation (co-IP)

In order to confirm the binding between SFMBT2 and HDAC3 or SIAH1, or determine the acetylation level, co-IP was performed. The cells were lysed with lysis buffer (Beyotime, Shanghai, China) supplemented with 1 mM PMSF. To determine the ubiquitination level of SFMBT2, the protein extraction was performed using denature lysis buffer (containing 50 mM Tris–Cl (pH6.8) and 2% SDS). The antibody was pre-incubated with the agarose gel beads, and incubated with the lysate at room temperature for 2 h. After washing, the antigen–antibody complex was used for SDS-PAGE. After electrophoresis, transfer and blocking, the protein was incubated with the antibody against SFMBT2 (1:1000; cat no. 25256-1-AP, Proteintech, Wuhan, Hubei, China), HDAC3 (1:5000; cat no. ab32369, Abcam, Cambridge, MA, USA), SIAH1 (1:300; cat no. sc-81785, Santa Cruz, CA, USA), ubiquitin (1:5000; cat. no. NB300-130SS, Novus Biologicals, Littleton, CO, USA), flag tag (1:1000; cat. no. AE005, ABclonal, Wuhan, China), or acetylated lysine (1:300; cat. no. sc-32268, Santa Cruz, CA, USA) at 4 °C overnight. Then the protein was incubated with corresponding secondary antibody, interacted with ECL reagent, and suffered with signal exposure in the dark.

### Methyl thiazolyl tetrazolium (MTT) assay

MTT assay was applied to measure the viability of cells. The cells were cultured in 96-well plates for a certain period of time, and treated with MTT reagent (50 μl per well) (KeyGene, Nanjing, China) for 4 h. Subsequence, the supernatant was removed, and the purple crystal was dissolved by dimethyl sulfoxide (DMSO) (150 μl per well). Finally, the optical density of the solution was assessed with a microplate reader (BioTek, Winooski, VT, USA) at 490 nm.

### Colony formation assay

The cells were inoculated in petri dishes with 200 cells per dish. After culture for about 2 weeks, the cells were fixed with 4% paraformaldehyde (Aladdin Biochemical Technology Co., Ltd., Shanghai, China) for 25 min, and stained with Giemsa reagent (KeyGene Biotech., Nanjing, China) for 5 min. The clones with more than 50 cells were counted.

### Flow cytometry

Flow cytometry was used for detection of cell cycle. The cells were collected and fixed with 70% ethanol at 4 °C overnight. Subsequently, the cells were stained with propidium iodide for 30 min in the dark, and the cell percentage in G1, S or G2 phases were determined with a flow cytometer (Agilent, Santa Clara, CA, USA).

### Wound healing assay

The wound healing assay was performed to detect the migratory ability of cells. The wound was made with a 200 μl pipette tip, and wound was photographed with a microscope (Olympus, Tokyo, Japan) at 100 × magnification after continuous culture for 0 h, 12 h or 24 h in serum-free medium. The wound size was determined, and the migration rate was calculated.

### Transwell assay

The transwell assay was executed to evaluate the invasive capacity of cells. The polycarbonate membrane of transwell chamber (Labselect, Hefei, Anhui, China) was pre-coated with matrigel (Corning, NY, USA) at 37 °C. The cells were inoculated into upper chamber with serum-free medium, and the lower chamber was infused with medium containing 10% FBS. After 24 h culture, the cells on the reverse surface of the membrane was fixed with 4% paraformaldehyde and stained with 0.5% crystal violet (Amresco, Solon, OH, USA). The cell numbers were counted under a microscope at 200 × magnification.

### HE staining

The tissue was immobilized with 4% paraformaldehyde overnight, and dehydrated with ethanol of grading concentrations (Sinopharm, Shanghai, China) and xylene (Aladdin Biochemical Technology Co., Ltd., Shanghai, China) for 30 min. Subsequently, the tissue was embedded with paraffin at 60 °C, and cut into 5 μm-sections, which underwent deparaffinization with xylene and ethanol. The sections were stained with hematoxylin (Solarbio, Beijing, China) for 5 min, soaked in 1% hydrochloric acid/ethanol for 3 s, and counterstained with eosin (Sangon Biotech (Shanghai) Co., Ltd., Shanghai, China) for 3 min. Finally, the sections were dehydrated, mounted with gum, and the images were acquired at 400 × magnification.

### Immunohistochemistry staining

The tissue was made into paraffin sections as described in HE staining section. After deparaffinization, the sections suffered from antigen retrieval in boiling for 10 min, and blocked with 3% H_2_O_2_ (Sinopharm, Shanghai, China) and 1% bovine serum albumin (Sangon Biotech (Shanghai) Co., Ltd., Shanghai, China) for 15 min, respectively. Then the sections were incubated with antibody against Ki-67 (1:100; cat. no. AF0198, Affinity, Changzhou, Jiangsu, China) in a humid box at 4 °C overnight in the dark. After rinsing with PBS, the sections were incubated with the secondary antibody at room temperature in the dark for 60 min, and interacted with DAB reagent (Solarbio, Beijing, China) for 5 min. Finally, the sections were stained with hematoxylin for 3 min and soaked in 1% hydrochloric acid/ethanol for 3 s, followed with dehydration, mounting and photographing at 400 × magnification.

The immunohistochemistry was evaluated via a combined score of the extent and intensity of staining [21]. Intensity was determined as 0 (no staining), 1 (weak), 2 (medium) and 3 (intensive). Extent was scored as 0 (no immunoreactive cells), 1 (1–25%), 2 (26–50%), 3 (51–75%) and 4 (75%). The final score was calculated by multiplying the score of extent and intensity (0–12). The SFMBT2 expression level was considered as high when the score was more than or equal to six, and as low when less than six. The scoring was performed by three pathologists in a blinded manner, and consensus was defined when at least two reviewers reached an agreement.

### Real-time PCR

Total RNA was extracted from cells with a TRIpure kit (BioTeke Corporation Co., Ltd., Beijing, China), and the concentration was assessed with an ultraviolet spectrophotometer. The RNA was reversely transcribed into cDNA using BeyoRT II M-MLV reverse transcriptase, in presence of oligo(dT) and random primer. The cDNA was applied for real-time PCR, and the program was set as follows: 95 °C for 5 min 10 s, 60 °C for 10 s, 72 °C for 15 s, followed with 40 cycles of 72 °C for 1 min 30 s, 40 °C for 1 min, melting 60–94 °C every 1 °C for 1 s, and finally incubation at 25 °C for 1–2 min. PCR was performed using the Exicycler™ 96 V4 Real-Time Quantitative Thermal Block (Bioneer, Deajeon, Korea). The data was calculated using 2^−ΔΔCT^ method. The sequences of real-time PCR primers were shown below:

- HDAC3 forward: 5′-TCGTCTTCAAGCCATACC-3′;
- HDAC3 reverse: 5′-ACACTGGGCAGTCATCG-3′;
- SFMBT2 forward: 5′-CTCTGCGAGGGAAAGGC-3′;
- SFMBT2 reverse: 5′-ACATAGCGAAGGCGTAA-3′.

### Animal experiments

Healthy 7-week-old male BALB/C nude mice were purchased from Cavens Experimental Animal Co., Ltd. (Changzhou, Jiangsu, China), and kept in a SPF environment (12 h/12 h light/dark cycles with 22 ± 1 °C and a humidity of 45–55%) with free access to food and water.

For xenograft model, the 786-O cells were subcutaneously inoculated into right flanks of mice (2 × 10^6^ per mouse). The tumor size was measured every 3 days. After 24 days, euthanasia was performed, and the tumors were isolated for subsequent detections.

For metastasis model, the 786-O cells with green fluorescent protein (GFP)-luciferase-lentivirus infection were injected into mice via tail vein (1 × 10^7^). Four weeks later, in vivo bioluminescence imaging was done using an animal imaging system (CLINX, Shanghai, China). Afterwards, the mice underwent euthanasia, and the lung metastasis was analyzed.

The feeding and experiments of animals were performed according to Guide for the Care and Use of Laboratory Animals, and approved by Ethics Committee of Shenyang Medical College (approval number: SYYXY2022102509).

### Statistical analysis

The data in this study were presented as mean ± SD, and analyzed with GraphPad Prism software. The data in different groups were analyzed using one-way or two-way ANOVA test, with Bonferroni’s multiple comparisons. The correlation between SFMBT2 expression in specimens and clinical information was analyzed with Chi-square test. A p value less than 0.05 was considered as significant.

## Results

### SFMBT2 was lowly expressed in advanced clear cell RCC specimens

**Fig. 1:**
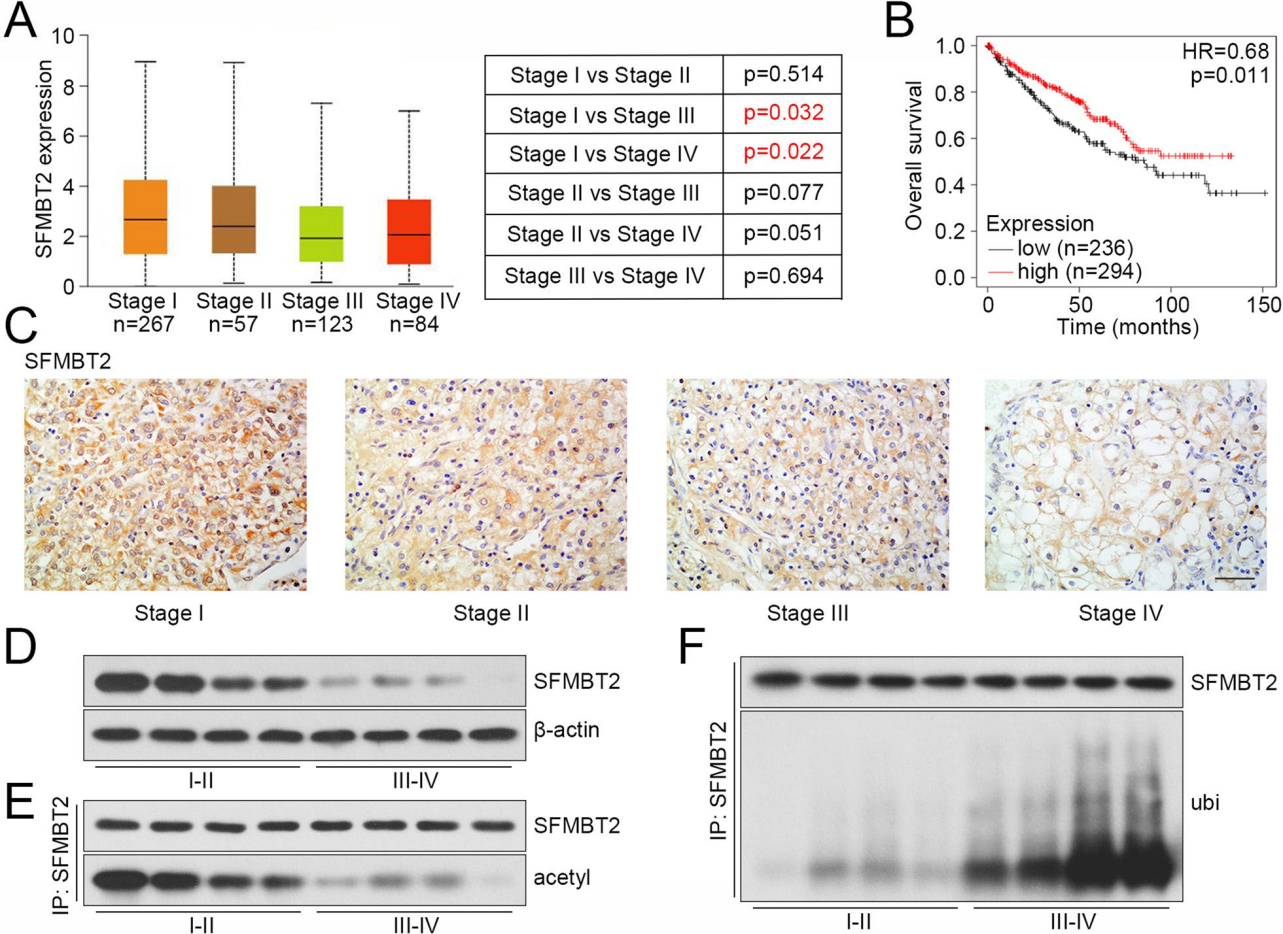
The expression of SFMBT2 in clear cell RCC clinical specimens. **A** The analysis of SFMBT2 in clear cell RCC specimens with different clinical stages from medical website UALCAN. **B** The survival analysis of clear cell RCC patients with different SFMBT2 expression from medical website Kaplan–Meier Plotter. **C** The expression of SFMBT2 in clear cell RCC specimens with different TNM stages was determined by immunohistochemistry, and the representative pictures were shown (the scale bar presented as 50 μm). **D** The SFMBT2 expression in clear cell RCC specimens with different stages. **E**, **F** The acetylation and ubiquitination levels of SFMBT2

The analysis from bioinformatic website UALCAN showed that SFMBT2 was significantly decreased in clear cell RCC specimens with clinical stage III/IV, compared with stage I or II (Fig. 1A). The survival analysis from Kaplan–Meier Plotter revealed that the clear cell RCC patients with SFMBT2 low expression possessed poor survival, comparing with those with SFMBT2 high expression (Fig. 1B). We collected sixty-one cases of clear cell RCC specimens, and determined the expression of SFMBT2. The analysis between SFMBT2 expression and clinical information revealed that SFMBT2 expression was related to TNM stage and T stage (Table 1). The clear cell RCC patients with more advanced clinical stage tended to have lower SFMBT2 expression levels. The representative images of SFMBT2 immunohistochemistry staining from clear cell RCC patients with grading stages were shown in Fig. 1C. In addition, the SFMBT2 expression and its acetylation/ubiquitination levels were determined with western blot and Co-IP in clear cell RCC specimens with different stages. As shown in Fig. 1D–F, the SFMBT2 was lowly expressed in specimens of stage III and IV, comparing with that of stage I and II, consistent with results from immunohistochemistry staining (Fig. 1D). The decreased acetylation level and increased ubiquitination level of SFMBT2 were observed in specimens of stage III and IV (Fig. 1E, F).

**Table 1:** The analysis between SFMBT2 expression and clinical information of clear cell RCC patients

Variable	Category	No. of cases		χ^2^	*p* value
		SFMBT2 Low	SFMBT2 High		
		n = 29	n = 32		
Age	≤ 60	17	20	0.01809	0.89300
	> 60	12	12		
Gender	Male	15	16	0.09593	0.75676
	Female	14	16		
TNM stage	I–II	14	25	5.87833	0.01533*
	III–IV	15	7		
T stage	T1–T2	14	26	7.32732	0.00679**
	T3–T4	15	6		

### SFMBT2 suppressed proliferation and growth of clear cell RCC cells

**Fig. 2:**
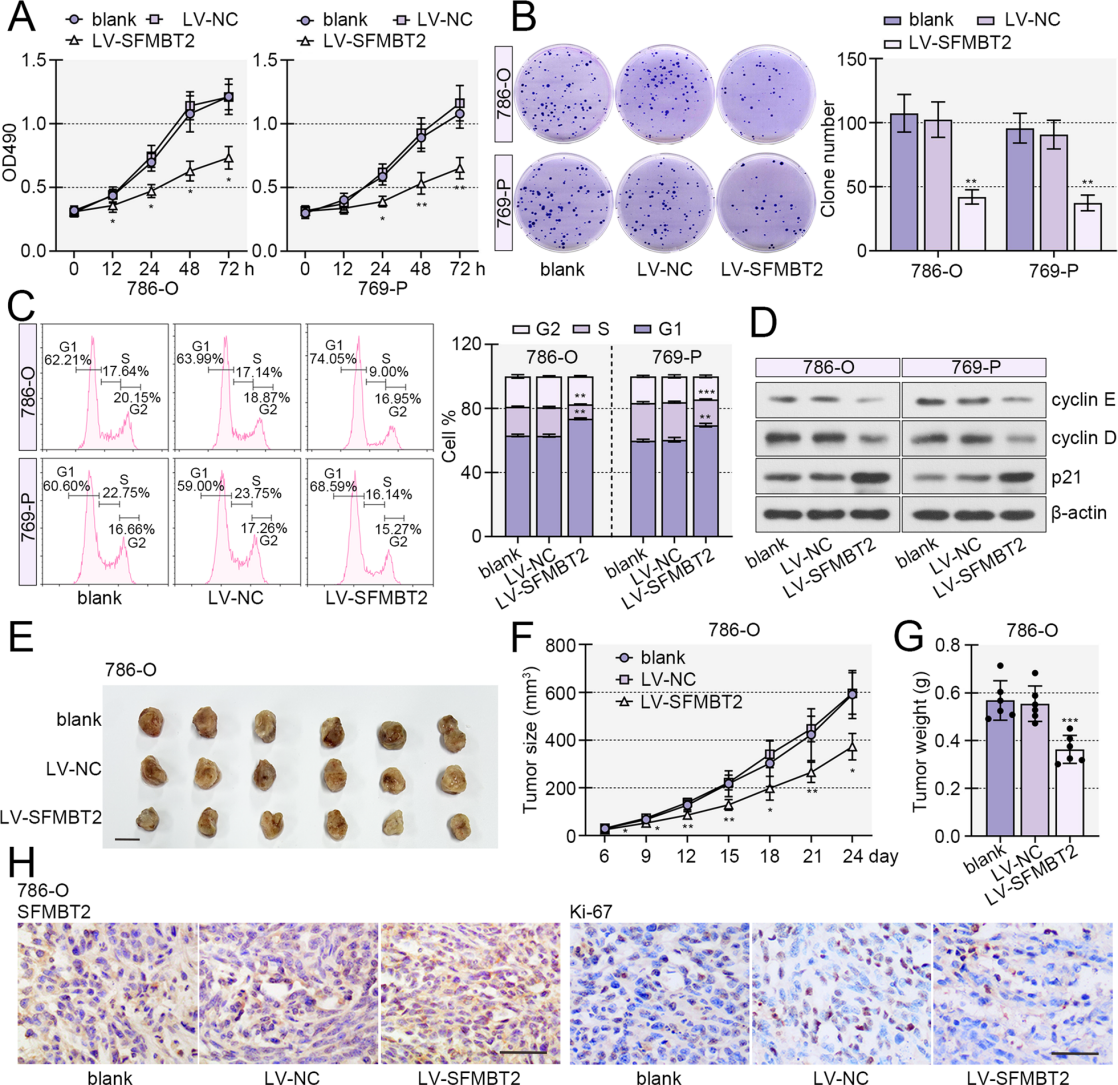
SFMBT2 suppressed proliferation and growth of clear cell RCC cells. **A** MTT assay was applied for measure the viability of clear cell RCC cells with SFMBT2 overexpression. **B** Plate colony formation assay was performed to assess the colony formation capacity of 786-O and 769-P cells. **C** The cell cycle was detected by flow cytometry. **D** Cell cycle-related protein, cyclin E, cyclin D and p21 were determined by western blot. **E** The 786-O cells with increased expression of SFMBT2 were inoculated into nude mice, and the subcutaneous tumors were shown (the scale bar presented as 1 cm). **F** The tumor growth curve. **G** The tumor weight. **H** Immunohistochemistry staining was performed to determine the expression of SFMBT2 and Ki-67 in the tumors (the scale bar presented as 50 μm). *p < 0.05;**p < 0.01; ***p < 0.001 versus LV-NC

**Fig. 3:**
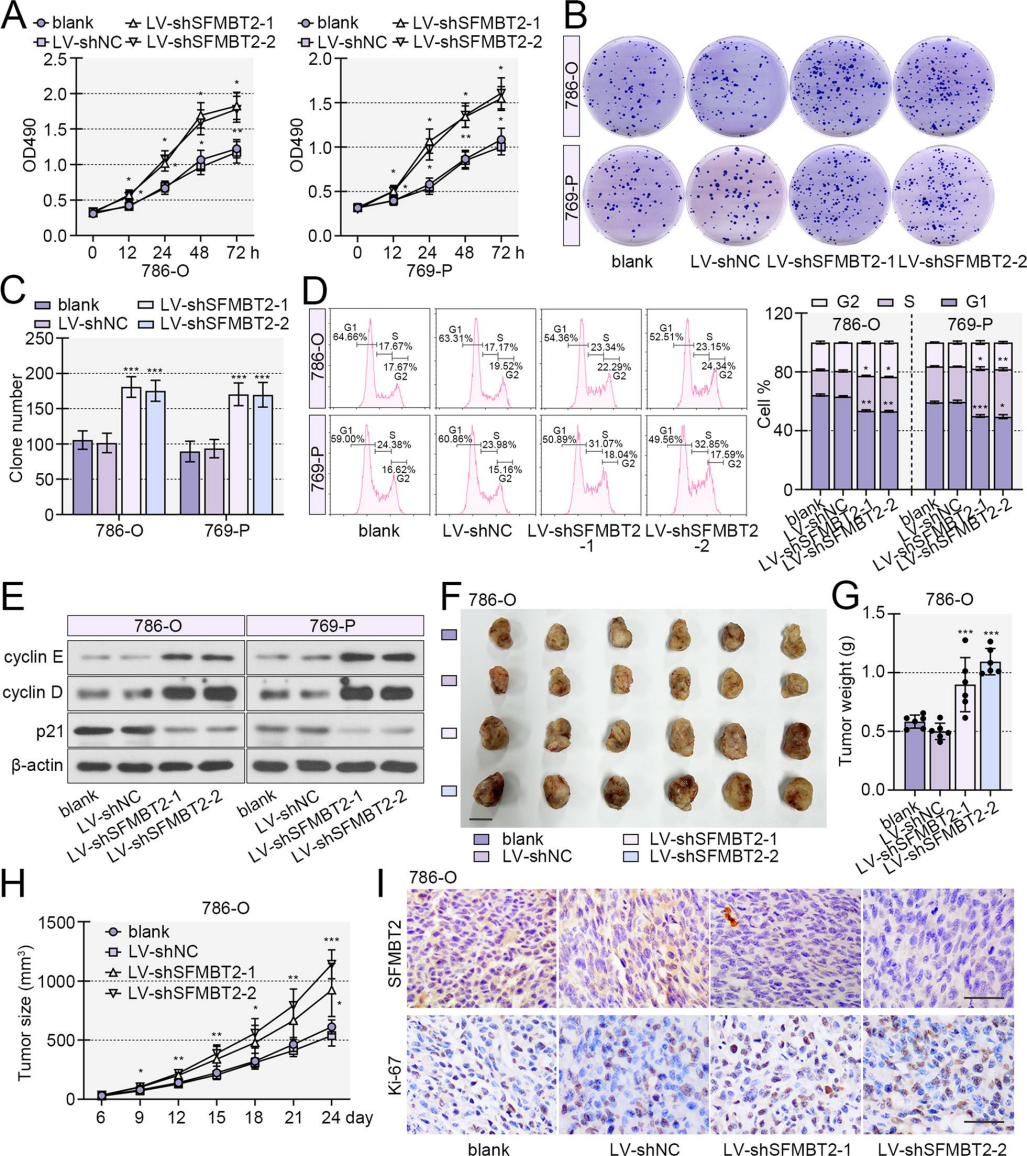
SFMBT2 knockdown facilitated proliferation and growth of clear cell RCC cells. **A** The cell ability was evaluated by MTT assay. **B**, **C** Plate colony formation assay was used to assess the colony formation capacity of 786-O and 769-P cells. **D** The cell percentage in phases. **E** The expression of cell cycle-related protein, cyclin E, cyclin D and p21 levels, was determined by western blot. **F** The 786-O cells with decreased expression of SFMBT2 were inoculated into nude mice, and the subcutaneous tumors were shown (the scale bar presented as 1 cm). **G** The tumor weight. **H** The tumor growth curve. **I** Immunohistochemistry staining was performed to determine the expression of SFMBT2 and Ki-67 in the tumors (the scale bar presented as 50 μm). *p < 0.05; **p < 0.01; ***p < 0.001 versus LV-shNC

To verify the role of SFMBT2 in clear cell RCC cells, lentivirus vector loading SFMBT2 coding sequence or silencing fragment was established and infected into clear cell RCC cell lines, 786-O and 769-P. The expression efficiency of SFMBT2 in 786-O and 769-P cells after lentivirus infection was confirmed by western blot (Additional file 1: Fig. S1). MTT and plated colony formation assays displayed that SFMBT2 overexpression suppressed the proliferation and colony formation of 786-O and 769-P cells, and its knockdown enhanced proliferation and colony formation (Figs. 2A, B, 3A–C). Flow cytometry and western blot revealed that enhanced expression of SFMBT2 delayed G1/S cell cycle transition, and its silencing accelerated cell cycle transition (Figs. 2C, D, 3D, E). The xenograft experiments revealed that ectopic expression of SFMBT2 facilitated the growth of 786-O cells in vivo, and the silencing of SFMBT2 played opposite roles (Figs. 2E–G, 3F–H). Immunohistochemistry staining demonstrated the SFMBT2 high expression in tumors from SFMBT2-overexpressed 786-O cells-inoculated mice, and its low expression in SFMBT2-silenced cells-inoculated mice (Figs. 2H, 3I). Meanwhile, SFMBT2-overexpressed tumors exhibited increased expression of Ki-67, a proliferation marker, and the SFMBT2-silenced tumors showed increased expression of Ki-67 (Figs. 2H, 3I). In addition, we also found that the acetylation and ubiquitination levels of SFMBT2 did not change in SFMBT2-overexpressed or -silenced tumors (Additional file 2: Fig. S2A-F).

### SFMBT2 inhibited migration, invasion and metastasis of clear cell RCC cells.

**Fig. 4:**
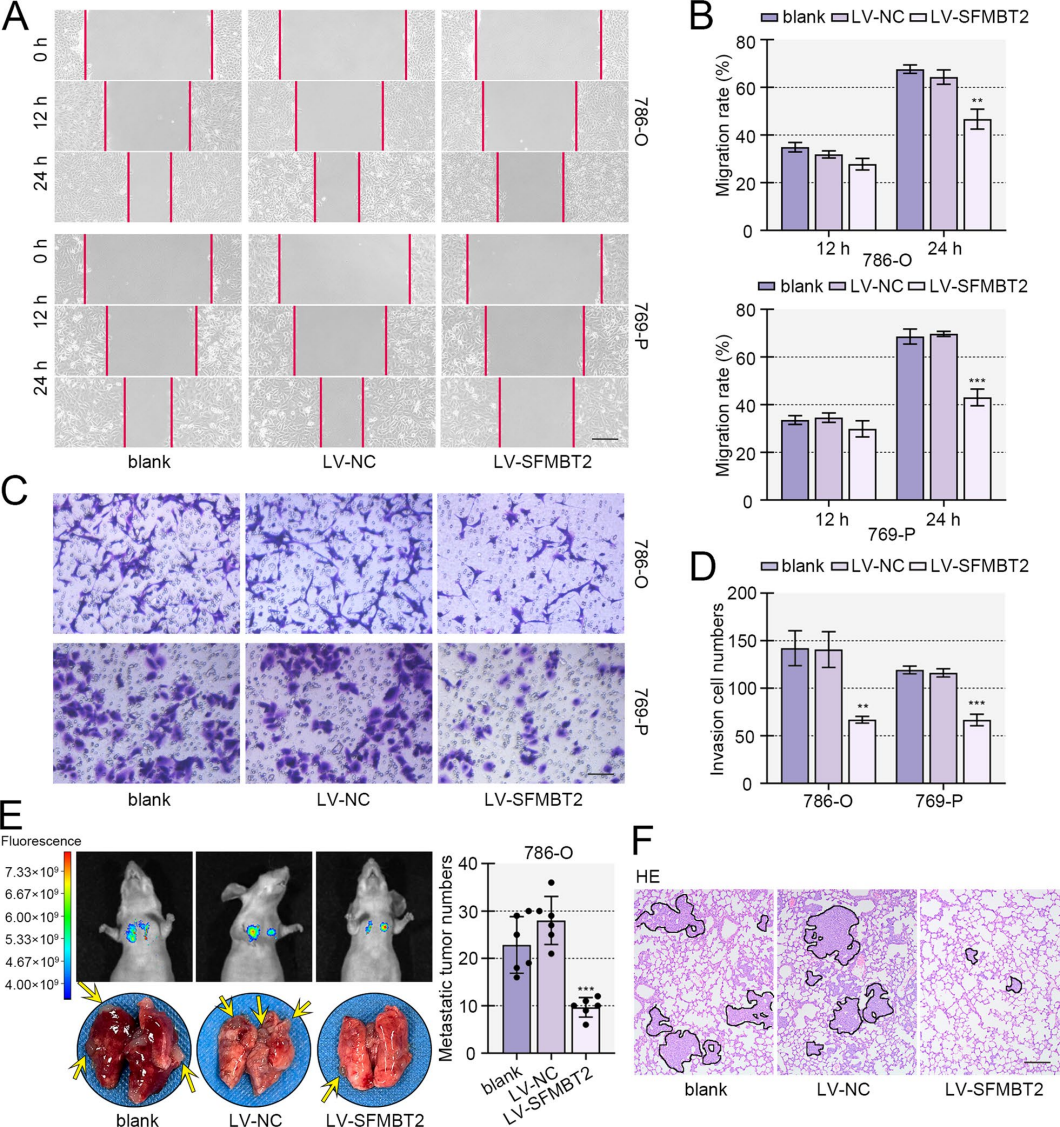
The enhanced expression of SFMBT2 inhibited migration, invasion and metastasis of clear cell RCC cells. **A**, **B** Wound healing assay was carried out to examine the migratory ability of clear cell RCC cells with SFMBT2 overexpression (the scale bar presented 200 μm). **C**, **D** Transwell assay supplemented with matrigel was used to detect the invasion of clear cell RCC cells (the scale bar presented 100 μm). **E** The GFP-stably-expressed 786-O cells were intravenously injected into nude mice, and the in vivo bioluminescence imaging was done. The lung was isolated from mice, and the nodule numbers in lung were counted. **F** HE staining was performed to measure the pathomorphological changes of lung (the scale bar presented 50 μm). *p < 0.05; **p < 0.01; ***p < 0.001 versus LV-NC

**Fig. 5:**
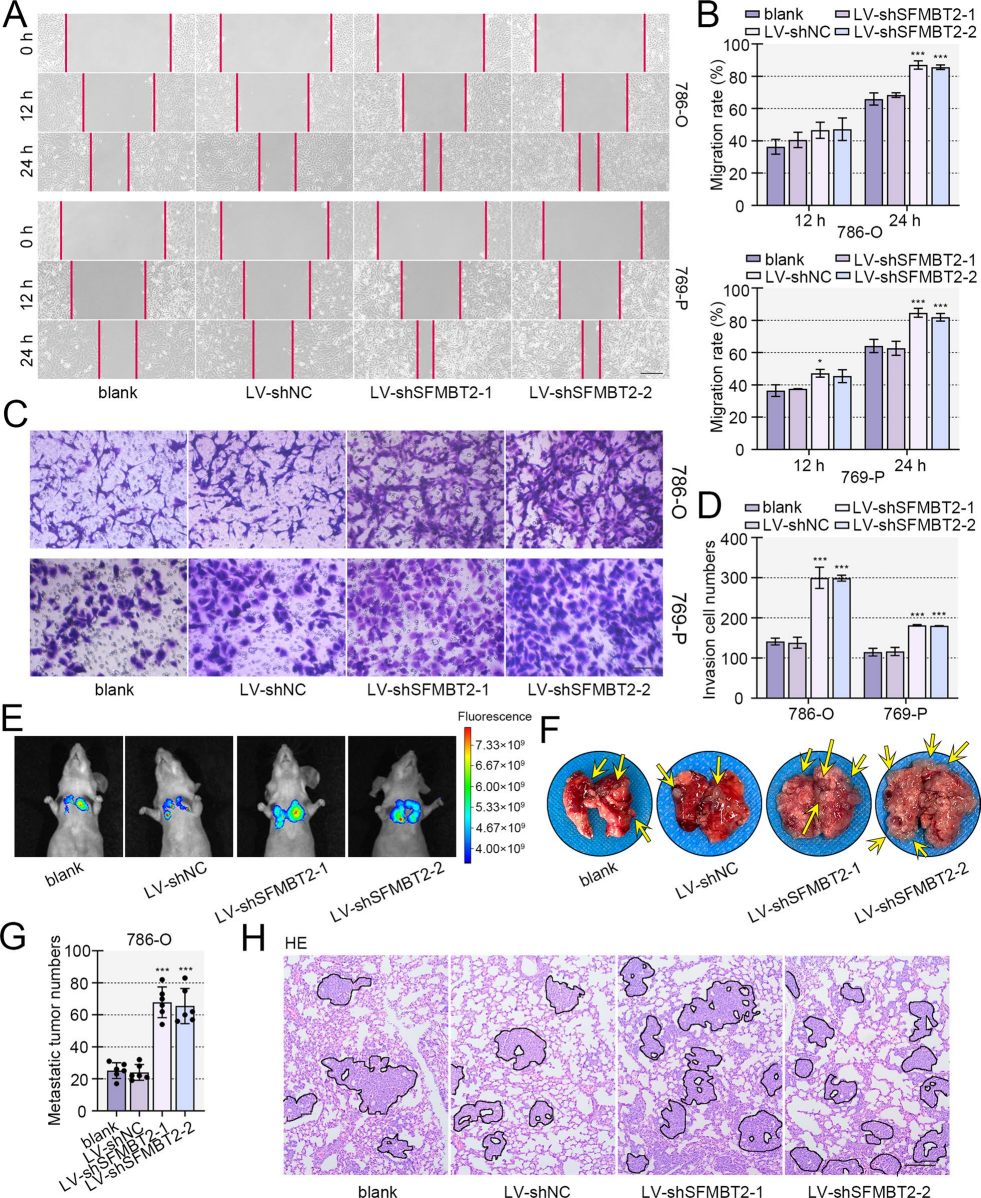
Silencing of SFMBT2 promoted the migration, invasion and metastasis of clear cell RCC cells. **A**, **B** The migratory ability of 786-O and 769-P cells was evaluated by wound healing assay (the scale bar presented 200 μm). **C**, **D** The invasion of cells were assessed by transwell assay with matrigel supplementary (the scale bar presented 100 μm). **E** The GFP-stably-expressed 786-O cells were intravenously injected into nude mice, and the in vivo bioluminescence imaging was done. **F**, **G** The lung was isolated from mice, and the nodule numbers in lung were counted. **H** The pathological features of lung from 786-O cell-inoculated mice were detected by HE staining (the scale bar presented 50 μm). *p < 0.05; **p < 0.01; ***p < 0.001 versus LV-shNC

Besides proliferation, metastasis is another cause of death for malignant tumors. Metastasis-related detections were performed. Wound healing assay revealed that enhanced expression of SFMBT2 induced migration retard, and SFMBT2 knockdown accelerated migration of 786-O and 769-P cells (Figs. 4A, B, 5A, B). Similarly, transwell assay displayed that infection of SFMBT2-expressed lentivirus led to decreased invasive ability, and SFMBT2 silencing exacerbated invasion of 786-O and 769-P cells (Figs. 4C, D, 5C, D). Results from metastasis experiment in vivo showed that the injection of 786-O cells induced tumor formation in lung of mice. SFMBT2 overexpression reduced the number and size of metastatic tumors, and SFBMT2 silencing promoted the tumor formation in lung (Figs. 4E, 5E–G). HE staining exhibited the tumors in lung (Figs. 4F, 5H).

### HDAC3 blocked SFMBT2 acetylation and accelerated its ubiquitination-degradation

**Fig. 6:**
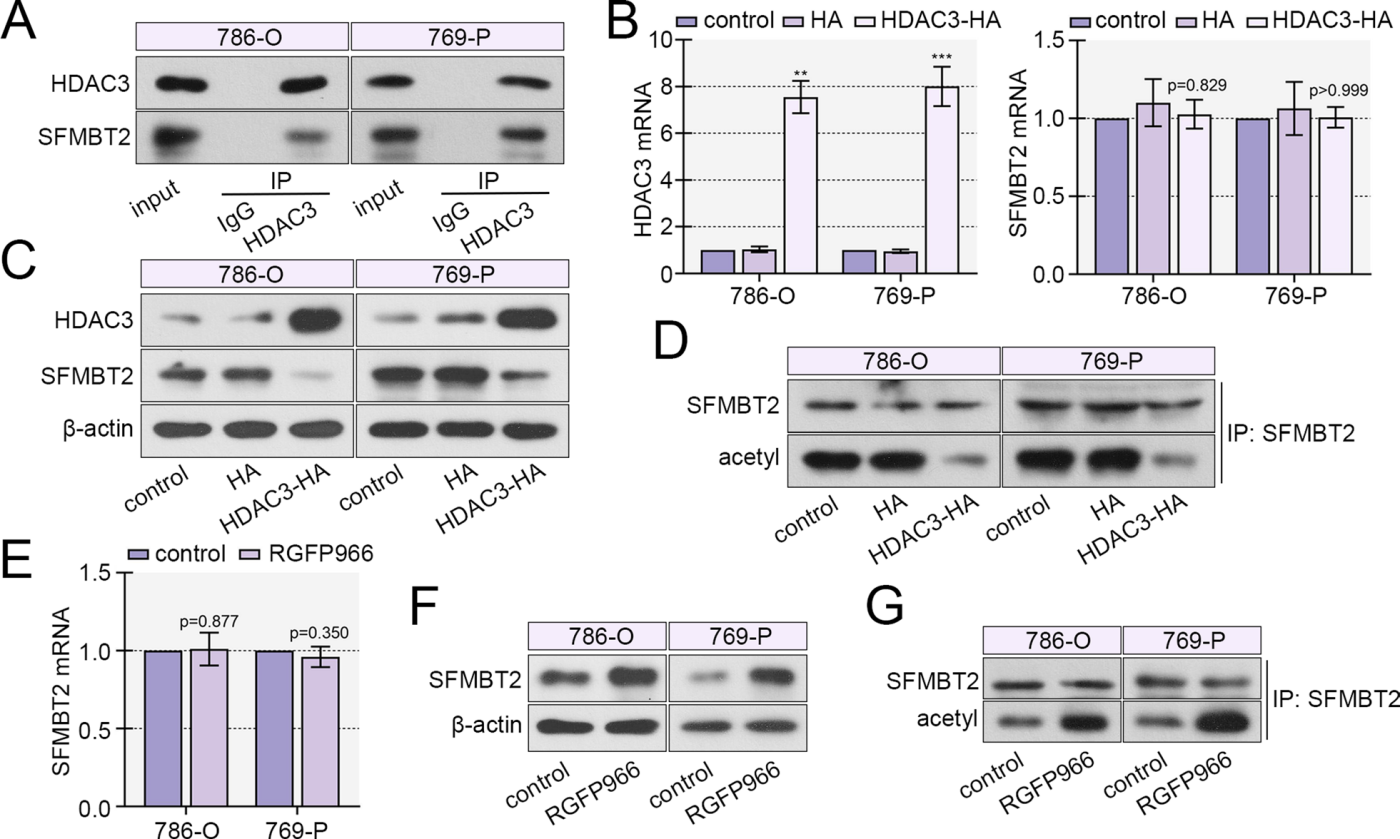
HDAC3 suppressed the protein level of SFMBT2 via deacetylation. **A** The binding between HDAC3 and SFMBT2 was confirmed by IP assay in clear cell RCC cells. **B**, **C** The HDAC3-overexpressed plasmid was transfected into clear cell RCC cells, the mRNA levels of HDAC3 and SFMBT2 were measured by real-time PCR, and their protein levels were examined by western blot. **D** The acetylated SFMBT2 level was determined by IP with SFMBT2 antibody incubation and acetylated lysine antibody blotting. **E**–**G** A HDAC3 inhibitor RGFP966 was used to treat the clear cell RCC cells, and the mRNA, protein and acetylated protein levels of SFMBT2 was determined. **p < 0.01; ***p < 0.001 versus HA

A previous article reported the binding between SFMBT2 and HDAC3 in prostate cancer cells. In our study, IP results confirmed their interaction in 786-O and 769-P cells (Fig. 6A). To investigate the function of HDAC3, an overexpression vector was constructed and transfected into clear cell RCC cells, and real-time PCR and western blot verified its expression efficiency (Fig. 6B, C). Subsequently, the results showed that HDAC3 overexpression reduced the protein level of SFMBT2, but did not affect its mRNA level (Fig. 6B, C). Meanwhile, HDAC3 decreased the acetylation level of SFMBT2 (Fig. 6D). Thereafter, a HDAC3 specific inhibitor, RGFP966, was applied. The data revealed that RGFP966 enhanced protein and acetylation levels of SFMBT2, but did not alter its mRNA level (Fig. 6E–G).

**Fig. 7:**
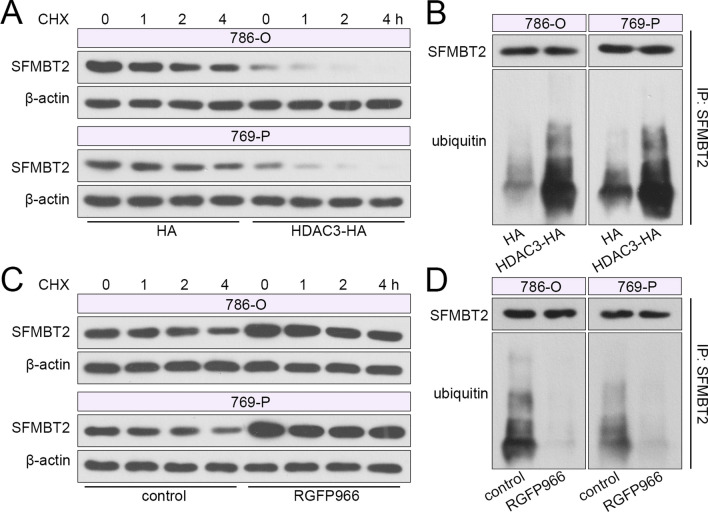
HDAC3 enhanced the ubiquitination and degradation of SFMBT2. **A** A translation inhibitor CHX was used in clear cell RCC cells for different times after ectopic expression of HDAC3, and the protein level of SFMBT2 was measured. **B** The ubiquitination level of SFMBT2 was detected after HDAC3 overexpression. **C** The protein level of SFMBT2 in clear cell RCC cells with administration of CHX and RGFP966. **D** The ubiquitination level of SFMBT2 after RGFP966 application

The degradation of SFMBT2 was determined in 786-O and 769-P cells via application of CHX, a translation inhibitor. The western blot results showed that after HDAC3 overexpression, the degradation of SFMBT2 was facilitated (Fig. 7A). IP results showed that HDAC3 promoted the ubiquitination level of SFMBT2 in 786-O and 769-P cells (Fig. 7B). Meanwhile, the inhibitor of HDAC3 delayed degradation and reduced ubiquitination of SFMBT2 in clear cell RCC cells (Fig. 7C, D).

### HDAC3 aggravated SIAH1-mediated ubiquitination of SFMBT2

**Fig. 8:**
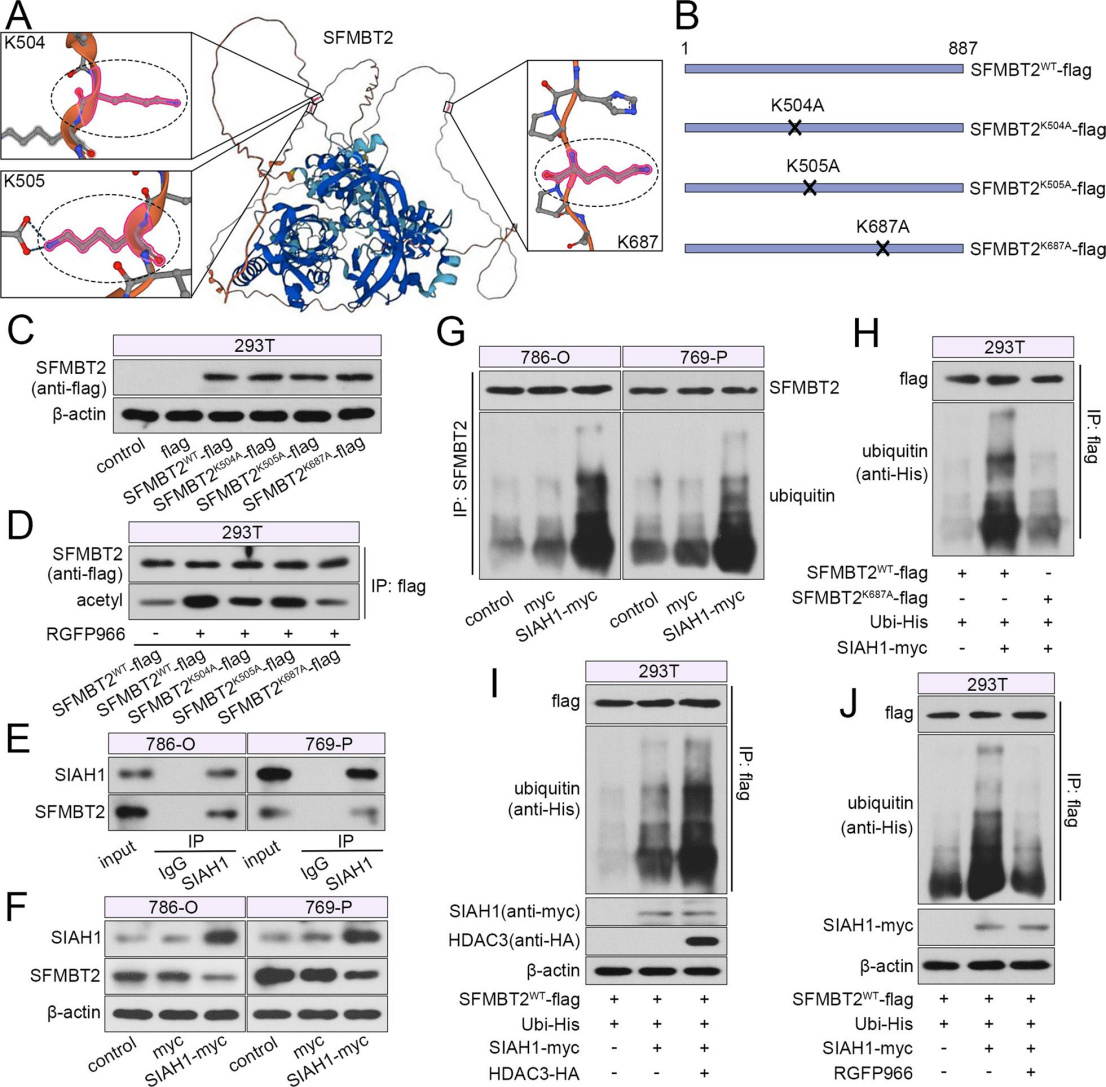
HDAC3 enhanced the ubiquitination of SFMBT2 mediated by SIAH1. **A** The structure of SFMBT2 protein and three potential acetylation/ubiquitination sites (K504, K505 and K687) from AlphaFold database (https://alphafold.ebi.ac.uk/) were shown. **B** Three SFMBT2 mutant vectors (K504A, K505A and K687A) were constructed. **C** These three mutant vectors were transfected into 293T cells, and the expression was verified by western blot. **D** The acetylated SFMBT2 levels were measured in 293T cells after RGFP966 treatment. **E** The binding between SIAH1 an SFMBT2 was confirmed by IP. **F** The SIAH1 expression plasmid was transfected into clear cell RCC cells, and the protein levels of SIAH1 and SFMBT1 were determined. **G** The ubiquitination levels of SFMBT2 after SIAH1 overexpression were detected. **H** The ubiquitination levels of SFMBT2 wild type or K687A mutant type were examined after SIAH1 overexpression. **I** The ubiquitination levels of SFMBT2 after overexpression of SIAH1 and HDAC3. **J** The ubiquitination levels of SFMBT2 in 293T cells with SIAH1 overexpression and RGFP966 application

Analysis from bioinformatic websites BioGRID and UbiBrowser (http://ubibrowser.bio-it.cn/ubibrowser_v3/) suggested three potential acetylation sites in SFMBT2 protein, K504, K505 and K687, as shown in Fig. 8A. To explore the regulation details of HDAC3 on SFMBT2, a WT and three acetylation-deficient mutant vectors (K504A, K505A, K687A) of SFMBT2 were constructed (Fig. 8B). Western blot demonstrated that these mutants did not affect the expression of SFMBT2 protein (Fig. 8C). Figure 8D showed that inhibition of HDAC3 significantly aggravated acetylation of WT SFMBT2, as well as K504A and K505A mutant molecules, but not K687A mutant (Fig. 8D), suggesting that K687 may be a key acetylation site of SFMBT2.

Since the binding of SFMBT2 with an E3 ligase SIAH1 was predicted, the function was investigated. IP results demonstrated the interaction between SFMBT2 and SIAH1 in 786-O and 769-P cells (Fig. 8E). Thereafter, SIAH1 overexpression vector was constructed and transfected into clear cell RCC cells. The high expression of SIAH1 decreased SFMBT2 protein levels, and enhanced its ubiquitination levels (Fig. 8F, G). Then the K687A SFMBT2 vector was used for IP, and the results revealed that SIAH1 only promoted the ubiquitination of WT SFMBT2, not K687A mutant SFMBT2 (Fig. 8H). Next, the crosstalk between acetylation and ubiquitination was verified. The IP results revealed that SIAH1-mediated ubiquitination of SFMBT2 was aggravated by HDAC3 overexpression, and attenuated by RGFP966 administration in 293T cells (Fig. 8I, J), suggesting that HDAC3 inhibitor-induced acetylation prevented SIAH1-mediated ubiquitination and degradation of SFMBT2.

## Discussion

In the present study, our data revealed that HDAC3 mediated deacetylation facilitated SFMBT2 degradation by accelerating SIAH1-mediated ubiquitination in clear cell RCC cells. The modification during gene expression classified into transcriptional modification, post-transcriptional modification, translational modification and post-translational modification, and the last one contains many types, such as ubiquitination, acetylation, phosphorylation, methylation, O-GlcNAcylation and hydroxylation [22]. These modifications result in the same protein having different conformations, locations, lifespans, activities or functions. Moreover, the crosstalk between these modifications makes the regulation of proteins more complex.

Ubiquitination is a major protein modification in mammals. The ubiquitin molecule binds to Lys residue (or Cys, Ser, Thr, Tyr residue) of substrate via covalent bond, and different ubiquitin linkage types endow substrate molecules with diverse alterations [20]. The typical K48 linkage mediates degradation of protein by proteasome, and may be affect by other modification. It has been reported that the phosphorylation of inhibitor of kappa B alpha (IκBα) on S32 and S36 is required for its ubiquitination [22]. RAR related orphan receptor A (RORα) with monomethylation is recognized and bound by DDB1-CUL4 E3 ubiquitin ligase [23]. p300-mediated acetylation of p53 attenuates its ubiquitination induced by mouse double minute 2 (MDM2) via inducing potential conformational changes [24]. Meanwhile, MDM2 functions by recruiting HDAC1 to deacetylate p53, and the ubiquitination and degradation are allowed [25]. However, increasing evidences suggest that the crosstalk between acetylation and ubiquitination may be not a single type. In some cases, the acetylation of a protein creates a high-affinity-binding site for other proteins. For example, it is known that hypoxia inducible factor 1 subunit alpha (HIF-1α) is rapidly degraded under normoxia through von Hippel-Lindau tumor suppressor (VHL)-mediated ubiquitination, and the N-alpha-acetyltransferase 10 (NAA10)-catalyzed acetylation enhanced the interaction of HIF-1α with VHL and its ubiquitination [26–28]. In our results, the acetylation of SFMBT2 prevented SIAH1-mediated ubiquitination and degradation, and HDAC3-catalyzed deacetylation enhanced its ubiquitination. This is consistent with the most common crosstalk between acetylation and ubiquitination. It was recently reported that the overexpression of HDAC3 promoted SIAH1-mediated ubiquitination of elongation factor for RNA polymerase II (ELL) in mammalian cells, similarly with our results.

Additionally, we demonstrated that K687 in SFMBT2 was the key site for acetylation and ubiquitination. The 3D structure of SFMBT2 protein molecule reveals that exposed K687 has a long protruding side chain, which may provide space for other molecules to approach, such as E3 ubiquitin ligase and acetyl. The other two potential sites, K504 and K505, are located inside the protein, and seems to be inaccessible to other molecule. The hydrophilic amidogen is essential for ubiquitination and acetylation. The mutant from Lys to Ala leads to the deficiency of amidogen and shorten of side chain [29]. However, the K687A, not K504A or K505A, resulted in significant reduction of acetylation and ubiquitination of SFMBT2, suggesting that the K687 residue was crucial for SIAH1-mediated ubiquitination and HDAC3 inhibition-induced acetylation.

Our data also demonstrated that SFMBT2 suppressed the growth and metastasis of clear cell RCC cells in vivo and in vitro. These results were consistent with a previous report, which demonstrated that SFMT2 inhibited migration and invasion of prostate cancer cell line LNCaP [10]. However, another paper showed opposite results, in which SFMBT2 promoted viability of prostate cancer cell line DU145 [12]. These contradictory results suggested that SFMBT2 may function differentially in various tissues or cell lines. SFMBT2 was demonstrated to act via regulating expression of other genes, so we supposed that the effect of SFMBT2 depended on function of downstream targets. For instance, SFMBT2 assisted in transcription suppression of MMP9 and MMP26, thereby inhibited metastasis of prostate cancer cells [10]. Decreased SFMBT2 contributed to degradation of extracellular matrix and blocking of chondrocyte proliferation in osteoarthritic cartilage by downregulating the expression of SOX9, which was essential for cartilage formation [30–32]. The function mechanism of SFMBT2 in clear cell RCC cells will be investigate in our next study.

SFMBT2 expression was downregulated in clear cell RCC patients with advanced stages, but it was highly expression in whole clear cell RCC specimens than paired para-carcinoma tissues (the data was not shown). It was an interesting appearance. In generally, we think that the excessive expression of oncogenes or deficiency of tumor-suppressing genes induces the tumorigenesis, such as well-known *myc*, *ras*, *TP53* and *PTEN* [33–35]. In this study, we hypothesized that SFMBT2 played a protective role in occurrence and progression of tumors. The expression of SFMBT2 increased after tumorigenesis, and the high levels of SFMBT2 attempted to curb tumor development. If SFMBT2 level was high enough, then the tumor was blocked at an early stage. If SFMBT2 level was not high enough, then the tumor continued to develop to an advanced stage. This hypothesis was supported by our experiments in cells and animals that SFMBT2 overexpression inhibited growth and metastasis of clear cell RCC cells in vitro and in vivo, and its knockdown exhibited opposite effects. However, this hypothesis needs to be verified by more clinical evidences.

The results from clinical specimens showed the downregulation of SFMBT2, as well as the decreased acetylation and increased ubiquitination. In xenograft tumors, the overexpression or knockdown of SFMBT2 did not affect its acetylation or ubiquitination level. Therefore, we speculated that the role of SFMBT2 depended only on its expression level. Ubiquitination and acetylation just function by affecting its protein level. On the other hand, neither HDAC3 nor SIAH1 could change the mRNA level of SFMBT2. However, the mRNA level of SFMBT2 was also decreased in clear cell RCC cases with advanced stages. We guessed that the expression of SFMBT2 was regulated at transcription or post-transcription level, and the details needed to be verified by more experiments.

In addition, the development of clear cell RCC has been reported to be associated with the dysregulation of lipid metabolism. Fatty acids are a major energy source for normal renal proximal tubule cells, and fatty acid oxidation in mitochondria supplies more than half of the ATP required to proximal tubular renal sodium reabsorption [36]. Obesity is identified as an independent risk factor for clear cell RCC, and it is associated with lipid accumulation in normal proximal tubule epithelial cells, which are the origination of clear cell RCC [37]. Clear cell RCC tissues are characterized by intracellular lipid accumulation, and several molecules have been reported to suppress the progress of clear cell RCC by regulating the lipid uptake and metabolism [38–40]. SFMBT2 was previously reported to inhibit the infiltration of preadipocytes in prostate cancer [11], suggesting that the function of SFMBT2 may be associated with the lipid metabolism. The details in clear cell RCC remain unknown. The correlation between SFMBT2 in lipid metabolism in RCC would be deeply investigated in our future.

## Conclusions

In the present study, we found that SFMBT2 was lowly expressed in clear cell RCC specimens with advanced stages. Overexpression of SFMBT2 suppressed growth and metastasis in vitro and in vivo of clear cell RCC cells. HDAC3-induced deacetylation enhanced SIAH1-mediated ubiquitination and degradation of SFMBT2, and K687 may be the key site. SFMBT2 may be considered as a novel clinical diagnostic marker and/or therapeutic target of clear cell RCC, and crosstalk between its post-translational modifications may provide novel insights for agent development.

### Supplementary Information

**Additional file 1**. **Figure S1**. The lentivirus loaded with SFMBT2 coding sequence or silencing fragment targeting SFMBT2 was delivered into clear cell RCC cells, 786-O and 769-P, and the efficiency was verified with western blot.

**Additional file 2**. **Figure S2**. The acetylation and ubiquitination levels of SFMBT2 in xenograft tumors did not change. The 786-O cells with SFMBT2 overexpression or knockdown were inoculated into nude mice, and the subcutaneous tumors were isolated. (A–C) The expression, acetylation and ubiquitination levels of SFMBT2 in tumors with ectopic expression of SFMBT2. (D–F) The expression, acetylation and ubiquitination levels of SFMBT2 in SFMBT2-silenced tumors.

## Data Availability

The data that support the findings of this study are available from the corresponding author upon reasonable request.

## Abbreviations

CHX: Cycloheximide
co-IP: Co-immunoprecipitation
DMSO: Dimethyl sulfoxide
ELL: Elongation factor for RNA polymerase II
FBS: Fetal bovine serum
GFPm: Green fluorescent protein
HDAC3: Histone deacetylase 3
HIF-1α: Hypoxia inducible factor 1 subunit alpha
HRP: Horse radish peroxidase
IκBα: Inhibitor of kappa B alpha
KATs: Lysine acetyltransferases
KDACs: Lysine deacetylases
MDM2: Mouse double minute 2
MMP: Matrix metalloproteinase
MTT: Methyl thiazolyl tetrazolium
NAA10: N-alpha-acetyltransferase 10
PcGs: Polycomb group proteins
RCC: Renal cell carcinoma
RORα: RAR related orphan receptor A
SFMBT2: Scm-like with four MBT domains protein 2
shRNA: Short hairpin RNA
SIAH1: Seven in absentia homolog 1
VHL: Von Hippel–Lindau tumor suppressor
WT: Wild type
