# Supplementary figures and images for: Acetylation- and ubiquitination-regulated SFMBT2 acts as a tumor suppressor in clear cell renal cell carcinoma
Source: Biol Direct. 2024 May 11;19:37. doi: 10.1186/s13062-024-00480-3 (PMC11088781; doi:10.1186/s13062-024-00480-3)

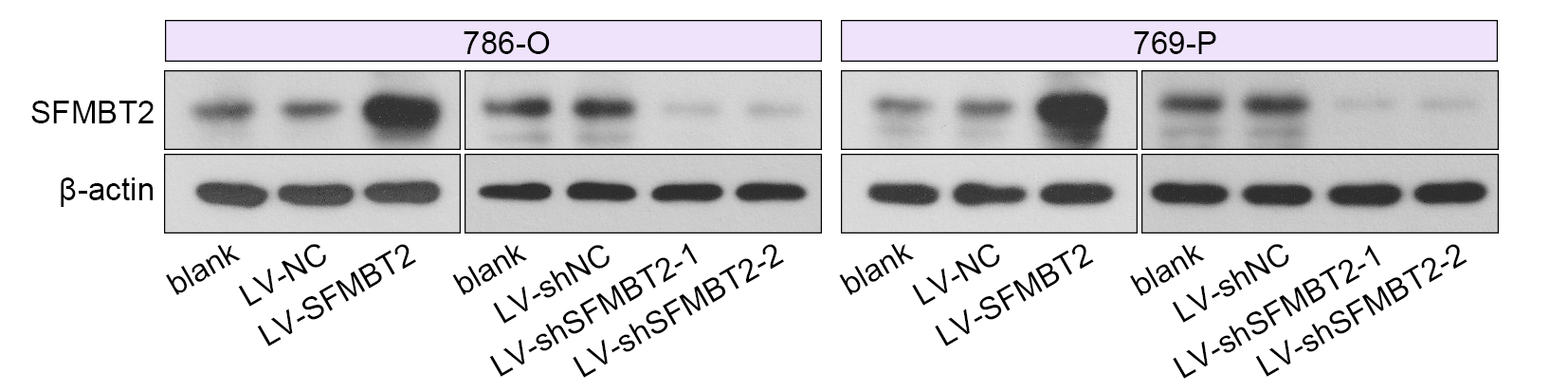

Supplement: Supplementary file 1 — Additional file 1. Figure S1. The lentivirus loaded with SFMBT2 coding sequence or silencing fragment targeting SFMBT2 was delivered into clear cell RCC cells, 786-O and 769-P, and the efficiency was verified with western blot. [file 13062_2024_480_MOESM1_ESM.tif]

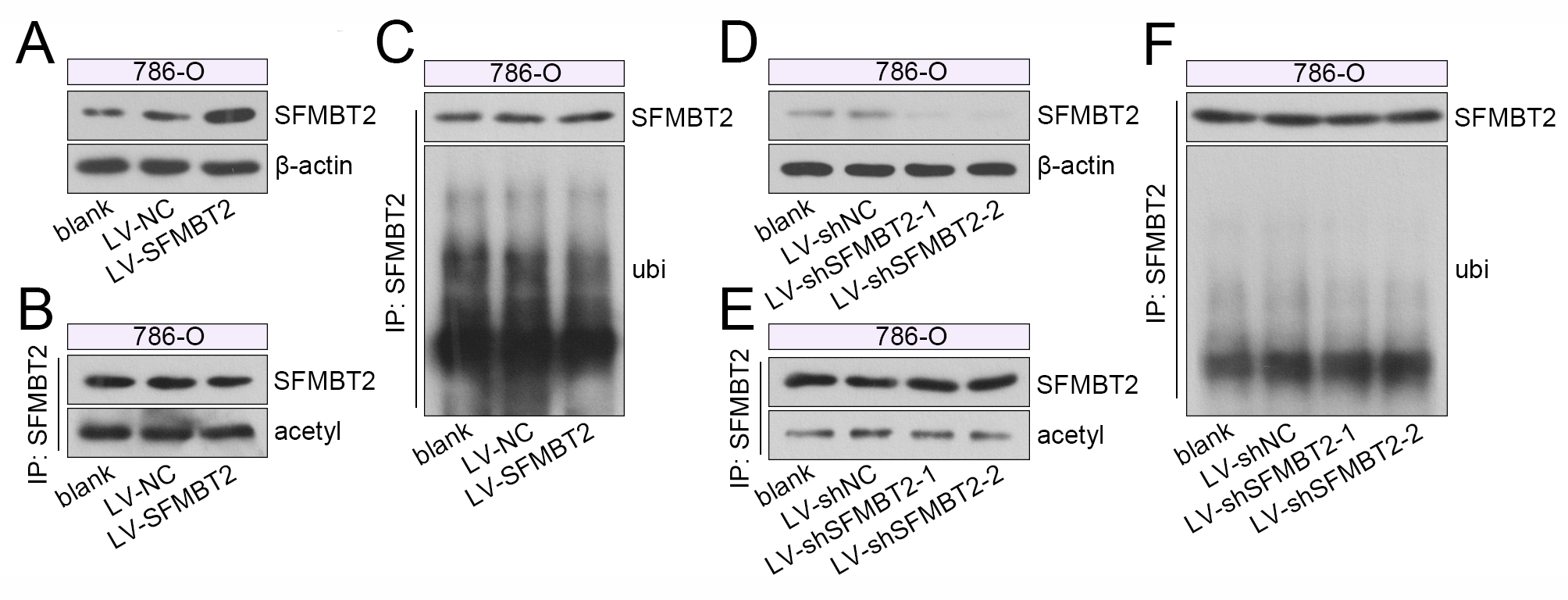

Supplement: Supplementary file 2 — Additional file 2. Figure S2. The acetylation and ubiquitination levels of SFMBT2 in xenograft tumors did not change. The 786-O cells with SFMBT2 overexpression or knockdown were inoculated into nude mice, and the subcutaneous tumors were isolated. (A–C) The expression, acetylation and ubiquitination levels of SFMBT2 in tumors with ectopic expression of SFMBT2. (D–F) The expression, acetylation and ubiquitination levels of SFMBT2 in SFMBT2-silenced tumors. [file 13062_2024_480_MOESM2_ESM.tif]
